# Retrospective evaluation of single patient investigational new drug (IND) requests in pediatric oncology

**DOI:** 10.1002/cam4.3791

**Published:** 2021-03-09

**Authors:** David S. Shulman, Lulla V. Kiwinda, Stacey Edwards, Catherine M. Clinton, Sarah Hunt, Lianne Greenspan, Kristen D. Lawler, Gregory Reaman, Hasan Al‐Sayegh, Kira Bona, Allison F. O'Neill, Suzanne Shusterman, Katherine A. Janeway, Andrew E. Place, Susan N. Chi, Clement Ma, Steven G. DuBois

**Affiliations:** ^1^ Dana‐Farber/Boston Children's Cancer and Blood Disorders Center Harvard Medical School Boston MA USA; ^2^ Boston Children's Hospital Boston MA USA; ^3^ US Food and Drug Administration Silver Spring MD USA; ^4^ Division of Population Sciences Dana‐Farber Cancer Institute Boston MA USA

**Keywords:** FDA, IND, investigational new drug, pediatric oncology, single patient IND

## Abstract

**Background:**

Single patient Investigational New Drug (IND) applications are one mechanism through which experimental therapies are accessed for children with cancer. The landscape of use, outcomes, and toxicity from single patient INDs remains unknown in pediatric oncology.

**Methods:**

We performed a retrospective analysis of all single patient INDs requested and prescribed at a single institution between 1/1/2007 and 5/1/2019. We report aggregate data from the US Food and Drug Administration (FDA) on single patient IND applications over the final two years of the study (2017–2019). We report an overview of all IND applications, as well as clinical descriptions of patients, treatments, outcomes, and toxicity.

**Results:**

Over the 2‐year period, the FDA approved all 171 submitted single patient IND requests for pediatric oncology. We identified 56 requests from our center during the 12‐year study period, and all were approved (median time from FDA submission to approval: 1 day (range 0–12)). 71% of requests were based on disease histology. Lack of pediatric clinical trial (65%) was the most common reason for use. 48 approved requests were ultimately administered. The median duration of treatment was 84 days (range: 4–1590), with 3 patients remaining on treatment at time of analysis. Only 7% discontinued treatment due to toxicity. Three‐year overall survival was 50% (95% CI, 35–64).

**Conclusions:**

Single patient INDs in pediatric oncology were universally approved in our national and single‐center analysis. In our cohort, single patient INDs were primarily utilized based on disease histology, rather than genomics, for agents that lacked a clinical trial.

## Graphical abstract

Single patient Investigational New Drug (IND) applications in pediatric oncology are universally approved in a timely fashion in our analysis. Single patient IND therapy is pursued for a range of reasons, with few patients discontinuing therapy due to toxicity.

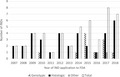

## 1 INTRODUCTION

Over the last 20 years, there has been a dramatic increase in the development of targeted anti‐cancer agents. The majority of new anti‐cancer agents are developed for cancers that primarily affect adults, and the median time from first in human testing to first in child testing is approximately 6.5 years.^1^ Further, only a small number of agents are ever granted a pediatric label indication. In recent years, the feasibility of identifying patients with somatic genomic alterations indicating sensitivity to novel agents has increased dramatically.^2^ This has led to the identification of children likely to benefit from targeted agents, but has left them with a lack of available clinical trial, commercially available approved agent, and/or available child‐friendly formulation. In the United States, these scenarios result in pursuit of single patient Investigational New Drug (IND) requests to the Food and Drug Administration (FDA) to obtain an unapproved agent under the FDA’s expanded access program. Although the recent Right to Try Act has raised concerns about barriers associated with the FDA’s expanded access program,^3^ the FDA receives roughly 1,000 applications per year and greater than 98% are approved.^4^, ^5^, ^6^, ^7^

The use of single patient INDs in oncology has been described primarily in adults with cancer. However, in one single institution study, children made up 34% of single patient INDs while only representing 2% of the overall patient population at the treating center.^8^ In a large survey of pediatric oncologists, over 50% had previously applied for a single patient IND and this was most common among clinicians who had practiced for greater than 15 years and those at large academic centers.^9^ There has not been a comprehensive assessment of the indications, toxicity, treatment strategies and outcomes for children with cancer being treated on single patient IND protocols.

As such, we performed a retrospective review of all single‐patient INDs at our large academic pediatric cancer center over the last 12 years. We evaluate the landscape of single‐patient INDs at our institution, including the breadth of agents requested, justification for use of specific agents and regulatory timelines. We report the toxicity and clinical outcomes of the patients who were treated with these agents. We also report the overall volume of single patient INDs submitted to the FDA for children with cancer during the final two years of our study.

## 2 PATIENTS AND METHODS

### 2.1 National evaluation of single patient IND requests to FDA

We queried internal US FDA databases to obtain the total number of pediatric (age < 18 years) single patient IND requests sent to the FDA between April 1^st^, 2017 and April 16^th^, 2019. We obtained data on the underlying disease group (solid/CNS tumor, hematologic malignancy) and whether an approval was granted.

### 2.2 Design and patient population for retrospective institutional study

We conducted a retrospective cohort study of single patient IND applications intended for use as anti‐cancer therapy in patients less than 30 years of age at Dana‐Farber/Boston Children's Cancer and Blood Disorders Center between January 1, 2007 and May 1, 2019, with a follow‐up data cut‐off of December 1^st^, 2019. Patients who received single patient INDs as supportive care medications were excluded. The start date was chosen to align with the start of the first full year of the use of the current Boston Children's Hospital electronic medical record. All patients and guardians provided informed consent for their initial single patient IND. This retrospective study was deemed exempt by the Dana‐Farber Cancer Institute Institutional Review Board in accordance with US federal policy for the protection of human subjects.

We performed a search of our clinical trial enrollment database and our Pediatric Patient Informatics Platform, both of which identify patients who were treated with single‐patient INDs. We also searched regulatory files going back to 2007 to identify patients treated with single patient INDs.

As a proxy for available pediatric dosing information, we searched clinicaltrials.gov and PubMed to determine whether a first‐in‐child trial was completed at the time of single patient IND start.

We included all single patient INDs that were filed with the FDA for an anti‐cancer agent. Patients treated as part of an expanded access protocol that allowed multiple patients to be enrolled were excluded (Table S1).

### 2.3 Variables

We performed a detailed review of the regulatory material associated with each IND as well as the electronic medical record to capture additional data for patients who met the defined criteria of having received treatment through a single‐patient IND. Data are reported in aggregate as each IND included specific requirements around publication that precluded reporting of patient‐level data for this cohort. For each IND, we captured the dates of FDA submission, IRB submission and approval/safe to proceed notification, whether the IND was filed for emergency use, the sponsor category of the IND, the formulation of the IND and the justification and reason for IND use. Of note, data on timing of submission, safe to proceed and whether emergency filing was necessary were only available for single patient INDs for which our institute was the sponsor. Clinical justification for use of the IND was coded as histologic, genotypic or other. For a patient to have a genotypic justification, they must have had a targetable genomic feature found on tumor profiling. Reasoning for pursuit of a single patient IND was coded as: pediatric formulation not available; no pediatric clinical trial; clinical trial accepts children but child unable to travel to study center; clinical trial available but child not eligible; or other. We collected information about diagnosis, stage of cancer at the time of treatment, prior clinical trial participation, and previously administered therapies. We also recorded the age, sex, race, ethnicity, primary language, and type of insurance (private/public‐only) of each patient. We collected dosing strategy, the duration of therapy, reason for discontinuation, as well as response as assessed using objective data by the treating clinician (radiologic/pathologic documentation and clinician notes). Detailed capture of all adverse events while receiving single patient IND therapy was outside the scope of this analysis. As a proxy for capturing serious adverse events, we recorded toxicities as events that met the following criteria: any unplanned clinic visits for toxicity (e.g., fever, need for transfusion); any emergency department visits; unplanned hospital admissions that were at least possibly related to study treatment; or transfer to the ICU for patients already admitted to the hospital. All data were stored in a secure internal REDCap database.

### 2.4 Statistical approach

Descriptive statistics (frequencies and proportions) were used to summarize the demographic, clinical, treatment, and outcome characteristics of the cohort. Fisher's exact test was used to test for an association between concurrent conventional chemotherapy and toxicity. No attempt was made to attribute toxicity to the study drug vs. the specific concurrent conventional chemotherapy agents. Overall survival (OS) was calculated from the beginning of IND treatment to the date of death, with patients without death censored at last follow‐up. Kaplan‐Meier curves of OS were generated. Two‐sided p‐values < 0.05 were considered statistically significant. Statistical analyses were performed using Stata^®^.

## 3 RESULTS

### 3.1 IND requests to the FDA for pediatric patients with cancer

From April 1^st^, 2017 through April 16^th^, 2019, there were 516 single patient IND requests to FDA for patients <18 years of age. Of these, 171 (33.1%) were for oncology indications. Twelve percent (n = 21) were for children with hematologic malignancies and 88% (n = 150) were for children with solid tumors or brain tumors. All requests for oncology indications were approved.

### 3.2 Requested single patient INDs at a single institution

**FIGURE 1:**
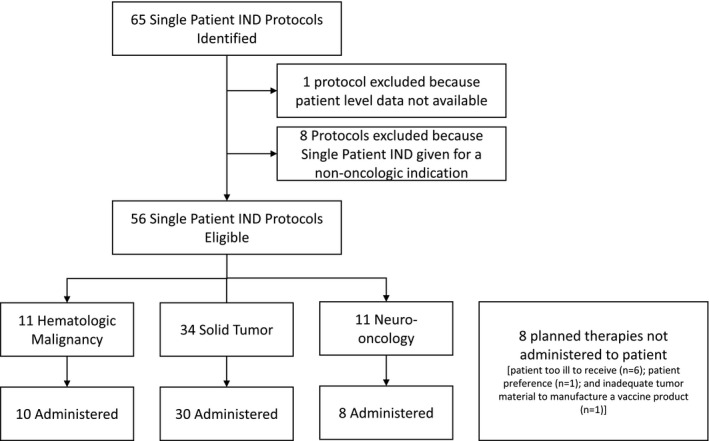
Consort diagram of single patient INDs identified during the study

Our local search returned 65 INDs submitted during the 12‐year study period. Eight INDs were excluded given the single patient IND was not for an oncologic diagnosis and one was excluded because patient level data were not available. Therefore, this analysis focused on 56 single patient INDs, all of which were approved by the FDA (Figure 1). The mechanisms of action of the included agents and underlying diseases relevant to these agents are described in Table S2. These single patient INDs were not part of a compassionate access protocol that allowed more than one patient to participate. Eight (14%) patients ultimately did not receive the investigational agent. The reasons patients did not receive the approved single patient IND included: patient too ill to receive (n = 6); patient preference (n = 1); and inadequate tumor material to manufacture a vaccine product (n = 1).

Details of the 56 approved single patient INDs are shown in Table 1. Small molecule inhibitors constituted the most common drug class. Our institution was most commonly the IND sponsor (n = 37). In 15 cases, procedures for submitting a single patient IND application through the NCI (n = 3) or the relevant pharmaceutical company (n = 12) had been developed and, in those instances, the relevant entity held the IND and was the sponsor. In total, 32 unique agents were requested. For the INDs for which our institution was the sponsor, the median duration from IND submission to safe to proceed notification from the FDA was 1 day (range: 0–12; n = 31). The median time from IRB submission to approval was 4 days (range: 1–16; n = 45). The rationale for request was most commonly based on disease histology (71%) and less commonly the disease genotype (24%). Among small molecule inhibitors, 50% were based on histologic justification and 50% were based on genotypic justification. Single patient INDs pursued based on genomic evidence were not necessarily obtained as part of precision oncology studies, but were pursued clinically based upon genomic data from sequencing results eligible for return for clinician use. Lack of available clinical trial was the most common regulatory reasoning for pursuing IND therapy (65%) and in 6% of cases there was no pediatric formulation of the agent otherwise available outside of a single patient IND.

**TABLE 1:** Characteristics of 56 single patient INDs

Characteristic	Frequency (%) Median (range)
Type of IND Agent	
Small Molecule	26 (46%)
Monoclonal antibody	9 (16%)
Other	21 (38%)
Type of Sponsor (n = 52)	
Institutional	37 (71%)
NCI	3 (6%)
Pharmaceutical	12 (23%)
Single patient INDs with emergency filing (n = 37)	5 (14%)
Time from FDA submission to safe to proceed (n = 31), days	1 (0–12)
Time from IRB submission to IRB approval (n = 45), days	4 (1–16)
Biologic justification for therapeutic approach (n = 55)	
Genotype‐based	13 (24%)
Histology‐based	39 (71%)
Other	3 (5%)
Regulatory reason for IND therapy (n = 54)	
Pediatric formulation not available	3 (6%)
No pediatric clinical trial available	35 (65%)
Clinical trial accepts children, but child unable to travel to study center	4 (7%)
Clinical trial available but patient not eligible	10 (18%)
Other	2 (4%)

### 3.3 Characteristics of patients treated with single‐patient INDs

**FIGURE 2:**
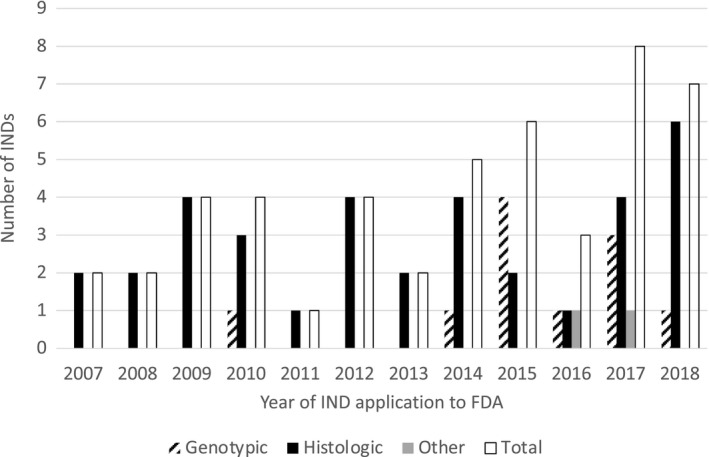
Number of patients treated per year on single patient INDs over time at our institution. Note that data from 2019 reflect partial year data and therefore not displayed

Ultimately, 48 single patient INDs were used at our center to treat the patient for whom an application was submitted. The number of patients treated on a single patient IND per year is shown in Figure 2.

The characteristics of the 48 patients treated on single patient INDs are shown in Table 2. The median age of patients treated with single patient INDs was 9.5 years (range: 0.1–28.5) and 40% were male. Eighty percent of patients identified as white, 4% black and 16% other. Among the overall population of patients treated at our center during the same time period, 77% identified as white, 6% as black, < 1% as American Indian or Alaska Native, 6% as Asian, and 11% as other. Twenty‐one percent (n = 10) of patients had a hematologic malignancy, 63% (n = 30) had an extracranial solid tumor, and 17% (n = 8) had a brain tumor. Twenty‐one percent (n = 10) of IND treatments were given as part of frontline therapy, and 79% were given for relapsed or progressive disease. Of the 35 patients with solid and CNS tumors who had available staging data, 23% had local or regional disease at time of treatment and 77% had metastatic disease.

**TABLE 2:** Characteristics of 48 patients treated on single patient INDs

Characteristic	Frequency (%) Median (range)
Age at IND treatment, median years (range)	9.5 (0.1 – 28.5)
Sex	
Male	19 (40%)
Female	29 (60%)
Race (n = 45)	
Black	2 (4%)
White	36 (80%)
Other	7 (16%)
Ethnicity (n = 46)	
Hispanic/Latino	8 (17%)
Non‐Hispanic/Latino	38 (83%)
Insurance at diagnosis (n = 46)	
Private	37 (80%)
Public	9 (20%)
Insurance at time of SPIND (n = 47)	
Private	36 (77%)
Public	11 (23%)
Primary Language	
Arabic	1 (2%)
English	44 (92%)
Spanish	3 (6%)
Disease Category	
Hematologic Malignancy	10 (21%)
Solid Tumor	30 (63%)
Brain Tumor	8 (17%)
Stage at IND Treatment (n = 35)	
Local/regional	8 (23%)
Metastatic	27 (77%)
Phase of therapy	
Frontline	10 (21%)
Relapse/Progression	38 (79%)
Prior lines of treatment	2.5 (0–12)
Prior clinical trial enrollment	
Yes	18 (37%)
No	30 (63%)
Prior Radiation	
Yes	22 (46%)
No	26 (54%)
Prior Stem Cell Transplant	
Yes	9 (19%)
No	39 (81%)
Prior Hospice Enrollment (n = 47)	
Yes	15 (32%)
No	32 (68%)

This cohort of patients had a median of 2.5 (range 0–12) prior lines of therapy, 37% (n = 18) of patients had previously enrolled on a clinical trial, 46% (n = 22) had prior radiotherapy, 19% (n = 9) had a prior autologous or allogeneic stem cell transplant, and 32% had enrolled previously in hospice services.

### 3.4 Characterization of single patient IND treatment

**FIGURE 3:**
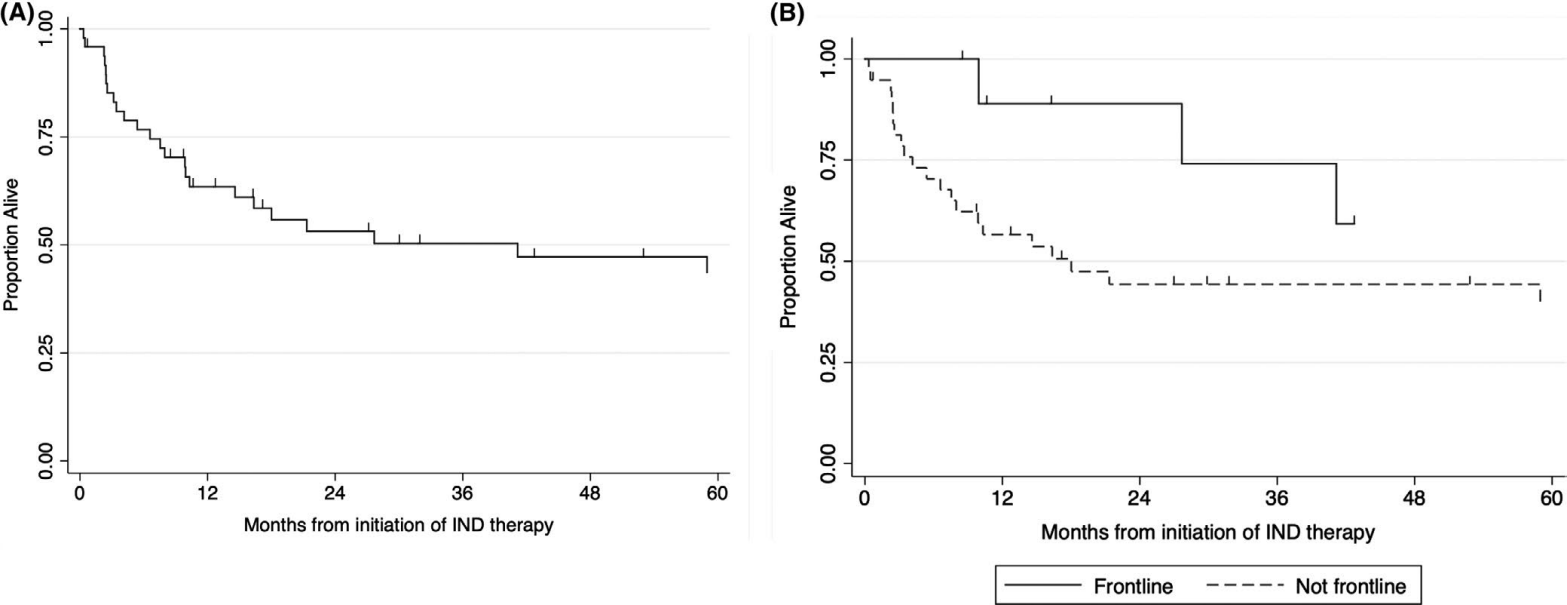
Overall survival for all 48 patients who received single patient INDs (A) and according to receipt as part of frontline vs. relapse therapy (B)

The median duration from FDA submission to treatment initiation was 24 days (range: 1–185; n = 27; Table 3). The median duration of treatment was 84 days (range: 4 – 1590) for the full cohort. Among patients receiving frontline therapy, the duration of therapy was 162 days (range: 13 – 1079) compared to 75 days (range: 4 – 1590) among patients with relapsed disease. Three patients remained on their IND therapy at the time of data review. Nine patients began IND therapy without evaluable disease, and one patient had inadequate clinical data available to assess response. Overall, 39% (15/38) of patients had a reduction in tumor burden and two patients had a complete response. One patient had a reduction in tumor burden before proceeding with definitive surgical resection. Three‐year overall survival was 50% (95% confidence interval [CI], 35–64) for the entire cohort, 74% (95% CI, 29–93; n = 10) for patients receiving frontline therapy, and 44% (95% CI, 28–60; n = 38) for patients receiving relapsed therapy (Figure 3A and 3B). Following completion of single patient IND therapy, 62.5% of patients went on to receive additional therapy. The median length of follow‐up among censored patients was 53 months (range 0.5–146 months).

**TABLE 3:** Treatment characteristics

Variable	Frequency (%) Median (range)
Time from FDA submission to drug administration INDs (n = 27), days	24 (1–185)
Given with conventional chemotherapy	
Yes	16 (33%)
No	32 (67%)
Duration of SPIND Use, days	84 (4‐1590^b^)
Frontline treatment duration (n = 10), days	162 (13–1079)
Relapse treatment duration (n = 38), days	75 (4–1590)
Reduction in disease burden or complete response (n = 38)^a^	15 (39%)
Complete response	2 (5%)
Dose modification required	12 (25%)
If dose mod required	
Decreased	7 (58%)
Increased	3 (25%)
Dose interruption	2 (17%)
Toxicity^c^	
Yes	26 (54%)
No	22 (46%)
Therapy discontinued at time of data analysis	
Yes	45 (94%)
No	3 (6%)
Reason for stopping SPIND therapy (n = 44)	
Toxicity	3 (7%)
Progression	20 (45%)
Completed Planned Cycle	13 (30%)
Other^d^	8 (18%)

^a^ 9 patients were not evaluable for response given lack of measurable disease at time of starting IND therapy and for one patient inadequate data was available to assess response.

^b^ 3 patients remain on therapy.

^c^ Unplanned Clinic visits for toxicity, ED visits for toxicity, and/or admissions/transfers to ICU for toxicity.

^d^ Other reasons for discontinuing therapy included: Tumor profiling revealed an alternative diagnosis (n = 2), patient received surgery with curative intent (n = 1), patient preference (n = 3), vaccine lot expired (n = 1), patient became eligible for a clinical trial (n = 1), patient death (n = 1).

At the time of single patient IND start, a first‐in‐child trial to inform pediatric dosing had been completed for 58% of agents. Overall, 54% of patients met the toxicity endpoint of having an unplanned clinic visit, unplanned admission, ICU transfer, or ED visit (Table 3). There was no difference in the rate of severe toxicity as assessed by our composite endpoint between patients who received concurrent conventional chemotherapy (56%; 9/16) compared to those who did not (53%; 17/32; *p* = 0.54). Dose modifications were required for 25% of patients, and 58% of those modifications were dose decreases. The reasons for discontinuing therapy included toxicity (7%), disease progression (45%), completion of planned treatment (30%), and other (18%).

## 4 DISCUSSION

We present the first comprehensive description of the use of single patient INDs for pediatric oncology patients in the era of targeted therapy. In our experience, single patient INDs were universally approved and were most commonly submitted for patients with relapsed extra‐cranial solid tumors (63%). By comparison, between January 1^st^, 2007 and December 31^st^, 2017, 34%, 35%, and 29% of patients with new diagnoses at our center were treated for hematologic malignancies, solid tumors or brain tumors, respectively.^10^ Interestingly, 21% of single patient IND therapies were administered as part of frontline therapy rather than for patients with relapsed disease. Many patients had an objective decrease in disease burden as assessed with radiographic scans and bone marrow biopsies, with 24 of 48 patients remaining on treatment for greater than 90 days. While toxicity was common, even as assessed through our composite endpoint, it was an uncommon reason for discontinuing therapy,

Whether the most appropriate means of accessing experimental medications outside of a clinical trial includes the FDA’s Expanded Access Program versus the Right To Try Act has been an ongoing debate in the literature and lay press.^3^, ^11^, ^12^ Much of the criticism of the Expanded Access Program stems from concerns that it is burdensome and prohibitive to patients receiving experimental, but potentially effective, medications. We present data from the FDA over the last two years (the only time period in our study in which aggregate national pediatric data were available) of this study showing that all pediatric oncology single patient INDs received were approved. Of single patient INDs submitted for children with cancer from our center over a 12‐year period, all applications were approved. In all cases in which a single patient IND was filed as an emergency filing due to the patient's clinical status, the IND was approved the same day or the following day. Further, for patients who received IND therapy, the median time from FDA submission to administration of the IND agent was 25 days, indicating that for most patients, the time required to obtain FDA safe to proceed was negligible in comparison to other steps required to administer therapy. This is similar to the regulatory timeline of 19 days reported previously in another series of single patient INDs for adults and children.^8^ In one emergency instance at our center, single patient IND therapy was administered one day following IND filing. While this study did not assess the burden to health care providers and regulatory staff, in our experience, IND filing is neither prohibitive with regard to access to specific agents or time required to obtain safe to proceed.

The growing understanding of molecular drivers of cancer and the rapidly expanding use of patient‐level tumor sequencing has resulted in cases in which clinicians identify genomic alterations that may indicate sensitivity to a targeted agent. For example, in recent years targetable fusions have been identified with high frequency in select pediatric tumors (i.e. NTRK‐fusions in infantile fibrosarcoma and mesoblastic nephromas or ALK/ROS1/NTRK3 fusions in infantile myofibroblastic tumors).^13^, ^14^, ^15^, ^16^ Coupled with the development of highly selective kinase inhibitors, there are a growing number of instances where there is strong clinical evidence for these inhibitors in children in spite of a lack of access to an approved drug or formulation.^17^, ^18^, ^19^ Interestingly, in our cohort, only 24% of single patient INDs were based on a targetable genomic finding, though these were enriched in later years of the study. It is unclear whether the use of single patient INDs to access genomically‐targeted therapies will increase with new drug development and more comprehensive tumor profiling, or decrease as children are increasingly included in genomically‐defined, age‐ and histology‐agnostic early drug development programs. Further multi‐institutional study of genomically‐oriented single patient INDs will be important to better understand this changing landscape.

In our cohort, many patients derived benefit from single patient IND therapy. Thirty‐nine percent of patients had some reduction in tumor burden and many patients remained on therapy for a prolonged period of time. While many patients met our composite toxicity endpoint, only 7% of patients discontinued therapy due to toxicity. While tracking toxicity of patients on single patient INDs is incredibly important to early drug development, in an analysis of ~1,000 referenced commercial INDs, there were only two instances in which adverse events occurring on a single patient IND led to a pause in commercial development and no instances in which development was terminated.^4^

Our study has a number of limitations. This study is a single institution experience and our experience may not be representative of other centers. Further, the time course for IRB approval and agent administration is partially dependent on our regulatory framework and clinical research infrastructure. These timelines may vary significantly between centers. However, we have included national data from the FDA to substantiate one of our primary findings that the FDA universally approved these therapies. We also surveyed across all disease groups at our center over more than a decade in an attempt to provide a comprehensive overview of this practice. The toxicity and outcome data in this retrospective study are limited in that it was not feasible to review retrospectively all responses and toxicity using standardized criteria such as RECIST and CTCAE. Nevertheless, we reviewed the treating team's assessment of the objective data, duration of therapy, and overall survival. To assess toxicity, we set a low bar for capturing clinically significant clinical events using our composite toxicity endpoint. However, given that not all toxicities were captured, these results should be interpreted with caution.

In summary, we provide a comprehensive assessment of the use of single patient INDs for children with cancer at a large academic medical center. These therapies have been most commonly used for children with extra‐cranial solid tumors and in many cases resulted in a decrease in tumor burden and/or prolonged stable disease. In our 12‐year experience, filing INDs to obtain access to experimental medicines did not pose a barrier to making these agents available to the study population in a timely and safe manner. Ultimately, as the molecular drivers of pediatric cancers become increasingly well understood, clinical trials of targeted agents should be made more readily available to children likely to benefit from novel agents. Simultaneously, the FDA has taken steps to expedite their review process leading to more rapid FDA approval of promising agents.^20^ However, given the current median delay of 6.5 years between first‐in‐human trials and first‐in‐pediatric trials,^1^ the expanded access program will remain an important means of providing children with cancer access to novel therapies.

## CONFLICTS OF INTEREST

SGD has received fees for consulting and advisory board roles from Bayer and Loxo Oncology and has received travel expenses from Loxo Oncology, Roche, and Salarius. AFO has participated in an advisory board for Fennec Pharmaceuticals. KAJ has received fees for consulting, travel support or honoraria for speaking from Bayer, Roche, Foundation Medicine and Ipsen. AEP has received fees for consulting and advisory board roles from AbbVie and Novartis. SNC receives reimbursement for travel expense from Epizyme.

## AUTHOR CONTRIBUTIONS

David S. Shulman: Conceptualization, data curation, funding acquisition, investigation, methodology, writing ‐ original draft, and writing ‐ review and editing; Lulla V. Kiwinda: Data curation, project administration, and writing ‐ review and editing; Stacey Edwards: Data curation, project administration, and writing ‐ review and editing; Catherine S. Clinton: Data curation, project administration, and writing ‐ review and editing; Sarah Hunt: Data curation, project administration, and writing ‐ review and editing; Lianne Greenspan: Data curation, and writing ‐ review and editing; Kristen Lawler D.: Data curation, and writing ‐ review and editing; Gregory Reaman: Data curation, conceptualization, and writing ‐ review and editing; Hasan Al‐Sayegh: Statistical analysis, conceptualization, and writing ‐ review and editing; Kira Bona: Conceptualization, and writing ‐ review and editing; Suzanne Shusterman: Conceptualization, and writing ‐ review and editing; Katherine A. Janeway: Conceptualization, and writing ‐ review and editing; Andrew E. Place: Conceptualization, and writing ‐ review and editing; Susan N. Chi; Conceptualization, and writing ‐ review and editing; Clement Ma: Statistical analysis, conceptualization, and writing ‐ review and editing; Steven G. DuBois: Conceptualization, data curation, funding acquisition, investigation, methodology, project administration, supervision, writing—original draft, and writing—review and editing.

## ETHICAL APPROVAL

This retrospective study was deemed exempt by the Dana‐Farber/Harvard Cancer Center Institutional Review Board in accordance with US federal policy for the protection of human subjects.

## Supporting information

Table S1‐S2

## Data Availability

The data that support the findings of this study are available from the corresponding author upon reasonable request.
