# Supplementary material for: Retrospective evaluation of single patient investigational new drug (IND) requests in pediatric oncology
Source: Cancer Med. 2021 Mar 9;10(7):2310–8. doi: 10.1002/cam4.3791 (PMC7982629; doi:10.1002/cam4.3791)
Supplement: Supplementary file 1 — Table S1‐S2 [file CAM4-10-2310-s001.docx]

**Supplemental Table 1.** Available multi-patient compassionate access protocols open during study period.

| Drug Class |
| --- |
| Chimeric antigen receptor T-cell therapy |
| ALK inhibitor |
| I-131 Radiopharmaceutical |

**Supplemental Table 2.** Mechanism of action of agents requested via single patient IND.

| Drug Classes Administered to Patient | | |
| --- | --- | --- |
| Drug Class | **Diagnoses** | **Number of Patients in Drug Class** |
| Asparagine specific enzyme | B-Cell ALL | 6 |
| Immunomodulator | Osteosarcoma | 4 |
| Alkylator (complex) | Synovial sarcoma | 4 |
| Aurora kinase A inhibitor | Atypical teratoid/ rhabdoid tumor | 4 |
| Anti-GD2 antibody | Neuroblastoma | 3 |
| Microtubule inhibitor | Neuroblastoma | 2 |
| ALK inhibitor | Inflammatory myofibroblastic tumor; Neuroblastoma | 2 |
| BET inhibitor | Nut carcinoma | 2 |
| Personalized cancer vaccine | Hepatocellular carcinoma; High-grade glioma | 2 |
| Gamma secretase inhibitor | Glomus tumor; desmoid fibromatosis | 2 |
| RET inhibitor | Infantile myofibroma; Medullary carcinoma of thyroid | 2 |
| Antibody drug conjugate to CD33 | AML | 1 |
| Anti-interferon gamma antibody | HLH | 1 |
| BCL2 inhibitor | AML | 1 |
| Anti-CTLA-4 antibody | Melanoma | 1 |
| Alkylator | Osteosarcoma | 1 |
| PI3K inhibitor | Rhabdomyosarcoma | 1 |
| EZH2 inhibitor | Epithelioid sarcoma | 1 |
| Antibody drug conjugate to CD56 | MPNST | 1 |
| MDM2/MDMX inhibitor | Rhabdomyosarcoma | 1 |
| Receptor tyrosine kinase inhibitor | Synovial sarcoma | 1 |
| Anti-DLL1 antibody | Atypical teratoid/ rhabdoid tumor | 1 |
| Pan-RAF inhibitor | Astrocytoma | 1 |
| HDAC inhibitor | NUT carcinoma | 1 |
| ACVR1 inhibitor | High-grade glioma | 1 |
| Chimeric antigen receptor T-cell therapy | B-Cell ALL | 1 |
| Drug Classes Submitted but Not Given to Patient | | |
| Drug Class | **Diagnoses** | **Number of Patients in Drug Class** |
| Anti-interferon gamma antibody | HLH | 1 |
| Alkylator (complex) | Synovial sarcoma | 1 |
| Anti-IGF-1R monoclonal antibody | Unknown | 1 |
| ERK-1/2 inhibitor | Hepatocellular carcinoma | 1 |
| FGFR inhibitor | Osteosarcoma | 1 |
| Aurora kinase A inhibitor | Atypical teratoid/ rhabdoid tumor | 1 |
| BET inhibitor | Medulloblastoma | 1 |
| Personalized cancer vaccine | Diffuse intrinsic pontine glioma | 1 |
